# The production of *Necator americanus* larvae for use in experimental human infection

**DOI:** 10.1186/s13071-022-05371-y

**Published:** 2022-07-08

**Authors:** Paul R. Chapman, Stacey Llewellyn, Helen Jennings, Luke Becker, Paul Giacomin, Rodney McDougall, Jennifer Robson, Alex Loukas, James McCarthy

**Affiliations:** 1 Clinical Tropical Medicine, QIMR Berghofer Medical Research Institute, Herston, QLD Australia; 2 Statistics Unit, QIMR Berghofer Medical Research Institute, Herston, QLD Australia; 3 Centre for Molecular Therapeutics, Australian Institute of Tropical Health and Medicine, James Cook University, Cairns, QLD Australia; 4 Sullivan Nicolaides Pathology, Bowen Hills and Herston, Australia; 5 RBWH Infectious Diseases, Bowen Hills and Herston, Australia

**Keywords:** Hookworm culture, Larval larvae, Hookworm larvae production

## Abstract

**Background:**

Although there is unprecedented interest in experimental human hookworm infection, details of hookworm manufacture and characterisation have been sparsely reported. In this report, we detail the production and characterisation of *Necator americanus* larvae for use in a recently published clinical trial.

**Methods:**

Faeces was obtained from an experimentally infected donor. Faecal hookworm DNA was determined by quantitative PCR. Paired samples were incubated in either sterile water or sterile water mixed with antimicrobials (amphotericin and gentamicin). Coproculture was performed by modified Harada-Mori method. The harvested larvae were then processed in either sterile water or antiseptic solution. Larval yield was then calculated (larvae per gram), larval viability was determined by thermally induced motility assay and microbial burden was determined at the day of harvest, at 48 h and at 7 days.

**Results:**

Twenty-eight faecal cultures were performed over 16 months. The faecal hookworm DNA content was variable over this time. There was no association of larval yield with faecal hookworm DNA content. Pre-treatment of faeces with antimicrobials did not influence larval yield. Larval motility was 85.3% (95% CI 79.3–91.3%). Incubation of larvae in antiseptics did not reduce viability at 14 days with a marginal mean of 68.6% (95% CI 59.1–78.1%) washed in water vs. 63.3% (95% CI 53.8 – 72.9%) when incubated in betadine (*p* = 0.38). Larvae washed in sterile water did not meet microbial bioburden criteria. Incubation in antiseptic resulted in acceptable microbial bioburden at 48 h but not at 7 days. Although the addition of gentamicin did reduce the microbial bio-burden acceptable levels, it was found to significantly lower larval motility at 7 days compared to incubation in sterile water and motility at 7 days 37.8% (95% CI 4.7–70.9%) vs. 67.3% (95% CI 35.2–99.3%, *p* < 0.001), respectively.

**Conclusions:**

Despite standardised culture methodologies and the use of a single donor, larval yield varied considerably between batches and had no association with faecal hookworm DNA. Larval viability decreases over time and the age of larvae at time of use are likely to be important. Microbial bioburden maybe temporarily reduced by incubation in antiseptics and has little effect on viability. Incubation of larvae in gentamicin is effective at reducing microbial bioburden but is deleterious to larval viability.

**Graphical Abstract:**

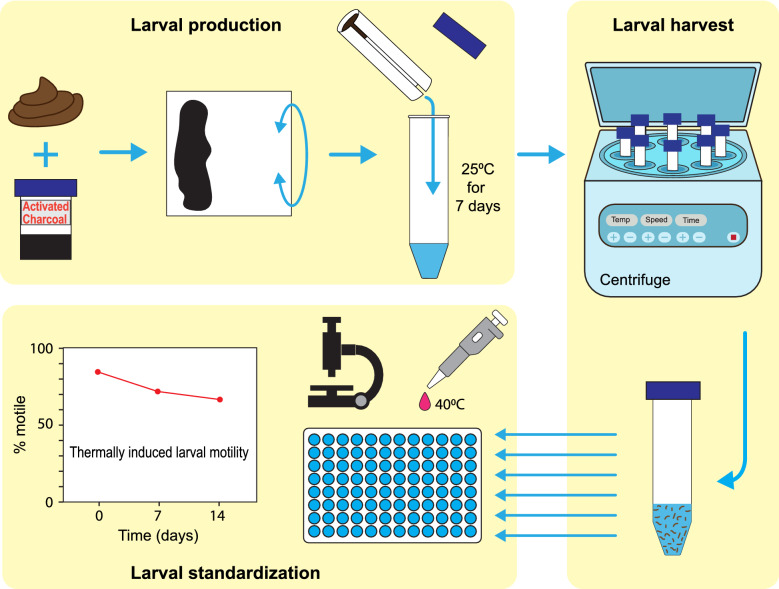

**Supplementary Information:**

The online version contains supplementary material available at 10.1186/s13071-022-05371-y.

## Background

Experimental human hookworm infection was first described in 1901 when Arthur Looss confirmed that *Necator americanus* larvae underwent dermal penetration to produce infection [1]. Experiments exploring the natural history of infection and immunological responses were performed throughout the twentieth century [2]. There is now unprecedented interest in experimental human hookworm infection, with recent trials published or registered to investigate the immunomodulatory potential of therapeutic hookworm infection (coeliac disease [3–5], inflammatory bowel disease [6–8], allergic rhinosinusitis and asthma [9, 10], multiple sclerosis[11] and as a cancer therapeutic [12]), challenge studies to investigate vaccines and therapeutic interventions [13–15] as well as reporting the safety and efficacy of controlled human hookworm infection in preparing human hookworm egg donors [16, 17].

In published human hookworm trials, methodology for preparing the larval inoculum has centred around modifications of the Harada and Mori method, first detailed in 1955 [18]. Although the use of larvae manufactured under current good manufacturing practice (cGMP) has been reported [11, 19], a detailed description of methods for larval culture and control of microbial contamination is lacking and is critical for regulatory purposes.

In this report we describe the development of larval culture methods, post-harvesting processes, viability testing and microbial bioburden testing for the production of *N. americanus* larvae used in a randomised clinical trial [15].

## Methods

Hookworm larvae were cultured, processed and stored in a dedicated PC2 laboratory at QIMR-Berghofer Medical Research Institute (Herston, Queensland, Australia) mimicking GMP principles. According to the Australian Therapeutic Goods Authority (the national regulatory agency), products used in Phase I tests do not need to be prepared to full GMP standards; further, challenge agents when not being used for “therapy” sensu stricto are exempt from these regulations. Laboratory safety procedures including personal protective equipment, larval handling and laboratory cleaning procedures were developed in accordance with published recommendations [20].

### Faecal culture

Faeces was sourced from a donor experimentally infected with *N. americanus* whose provenance were from a line of *N. americanus* originally sourced from Papua New Guinea by Prof. David Pritchard of the University of Nottingham and since maintained in experimentally infected human donors [21]. The donor was a 36-year-old male, inoculated with 25 larvae on 17 May 2016 in an associated human research study (Experimental low-dose infection of human volunteers with the hookworm *Necator americanus*) with human research and ethics approvals provided by James Cook University (approval number H5936). The donor was screened for transmissible blood-born viruses (HIV, HBV, HCV) and for infection with significant bacterial enteropathogens (Salmonella, Shigella, Campylobacter).

Faecal coproculture was established within 48 h of defecation. The culture method was a modification of the Harada-Mori method [18]. The faeces was homogenised by stirring, after which an aliquot was removed and stored at 2 °C for determination of hookworm DNA content by qPCR (as described below). The sample was then emulsified with activated charcoal (which balances pH and reduces the offensive odour) and sterile water until the consistency of the sample resembled bitumen or tar.

Approximately 7 g of the culture material was then smeared on to the upper third of 9 × 9-cm squares of 80gsm copy paper (Reflex Australian Paper, Mt Waverly, Australia) and rolled into cylinders (culture material innermost) and placed within 50-ml sterile centrifuge tubes (Corning, New York, USA) containing 5 ml sterile water. Copy paper degrades more slowly than the filter paper traditionally used, producing cleaner harvests. The base of the paper roll rests in the water, with the culture material near the top of the tube with the lid loosely secured. Approximately 20 tubes were produced per 100 g of faeces. These were then stored in a tube rack placed within a plastic box. To maintain a humid environment, the base of the box was lined with adsorbent paper soaked in water. The boxes were then placed in the incubator at 25 °C for 7 days.

#### Faecal hookworm DNA qPCR

Assessment of faecal hookworm DNA content by qPCR was performed using a variation of previously described methods [22], in either duplex assay with *Necator* sp. and the equine herpes virus (EHV) extraction control or for initial samples as a four-plex assay additionally testing *Ancylostoma* sp. and *Ascaris* sp. Further details on qPCR methodology and DNA extraction are provided in Additional file 1: Methods S1.

### Preculture antibiotic treatment

To determine whether pre-treatment of faeces with antimicrobials affects the larval yield or microbial burden, parallel cultures were prepared with either (i) amphotericin B deoxycholate (15 mg/100 g faeces) and gentamicin (60 mg/100 g faeces) and sterile water or (ii) sterile water only. The faeces were incubated with the antibiotics for at least 1 h before the addition of activated charcoal (which is likely to inactivate free antibiotic [23]).

### Larval harvest and determining larval yield

To harvest the larvae the roll of paper was carefully removed and discarded from each tube. The remaining fluid containing the larvae was then combined into 50 ml sterile centrifuge tubes (Corning, New York, USA) and centrifuged at 2000 *g* for 5 min with the brake off. Forty-five milliliters of supernatant was then discarded and the larvae were resuspended in sterile water. This washing process was performed three times before resuspension of the larvae in 2 ml of sterile water in a 15-ml conical tube. Larval yields were calculated by examining the number of larvae in 5 × 20 µl aliquots of resuspended larval solution at × 40 magnification.

### Post-culture processing and estimation of microbial bioburden

A set of experiments was performed to determine the microbial bioburden of the larval solution and the effect of incubation in betadine (povidone-iodine solution, 10% w/v, Sanofi consumer healthcare, Virginia, Australia) and of storage in gentamicin (Pfizer, Melbourne, Australia)-containing solution. After harvest, the larval solution was incubated for 10 min at room temperature with an equal volume of betadine at a concentration of 1%, 0.1% and 0.01% equivalent iodine. The solution was then centrifuged, supernatant discarded and larvae washed and resuspended in sterile water three times. The larvae were then stored in 15-ml centrifuge tubes suspended in either sterile water or sterile water with 1, 0.1, 0.5, 0.01 or 0.001 mg/ml gentamicin.

### Estimation of microbial bioburden

Aliquots (100 µl) of hookworm supernatant (the volume in which larval dose was suspended for the clinical trial) were analysed for microbial bioburden immediately post-harvest, at 48 h and at 7 days following processing.

As there is no regulatory guidance by the Australian Therapeutic Goods Administration (TGA) for microbial quality for dermally applied helminth larvae, criteria were therefore adapted from “TGA, Guidance for Microbial Quality Criteria for Transdermal patches Sect. 17.3.1” [24], criteria analogous to the United States Pharmacopeia criteria 61 and 62: microbial examination of non-sterile products [25]. Total aerobic microbial count and total yeast and mould count and the presence or absence of “objectionable organisms” (*Staphylococcus aureus*, beta-haemolytic streptococci and *Pseudomonas aeruginosa*) were determined according to A.P.H.A Standard Method 9215C [26]. Although beta-haemolytic streptococci are not included in the TGA guidance, this species was included as an objectionable organism because of its unquestionable dermal pathogenicity.

### Larval viability

Larval viability was estimated by thermally induced motility assay as described previously [27]. Triplicate samples of 20–30 larvae, suspended in 100 ul sterile water, were loaded into a 96-well plate. Motility was observed at × 40 magnification following the addition of 50 ul of 40 °C water to the well. Viability was recorded as the percentage of motile larvae (motile larvae/total larvae in well × 100).

### Statistical analysis

Normally distributed continuous measures were summarised by mean and standard deviation (SD) or 95% confidence interval (CI), and non-normally distributed continuous measures were summarised by median and interquartile range (IQR). Larval yield per gram of faeces was compared between gentamicin treatment groups using a Mann-Whitney test for non-normally distributed data. Associations between non-normally distributed continuous variables, including larval yield, PCR Cq results and culture date, were assessed using Spearman’s rank order correlation. Associations between larval motility and betadine or gentamicin treatment groups were assessed using linear mixed effects models with random intercept for larval batch to account for batch variability in motility. Statistical analyses were performed in SPSS version 23 (IBM Corp, Armonk, NY) and R statistical package version 4.0.2.

## Results

Twenty-eight cultures were performed using 28 individual faeces samples between 4 July 2017 and 12 November 2018 (16 months). Donor faecal hookworm qPCR Cq value was variable over the 16 months (Additional file 1: Fig. S1A), with some evidence of a negative association in qPCR Cq value over time (ρ = − 0.42, *p* = 0.025, lower Cq value indicates a larger amount of DNA present; Additional file 1: Fig. S1B); however, there was no significant correlation between time and larval yield. There was no evidence of an association between larval yield per gram (LPG) and qPCR Cq value (ρ = 0.14, *p* = 0.48) Additional file 1: Fig. S1).

Pre-treatment of the faeces with amphotericin B and gentamicin was not found to influence LPG with median (IQR) of 26.9.0 (5.7–74.7) for the 16 treated cultures vs. 18.30 (6.4–39.6) for the 12 untreated cultures (*p* = 0.16).

Mean larval motility following harvest was 85.3% (95% CI 79.3–91.3%). Compared to washing in sterile water, incubating larvae in 1% betadine for 10 min prior to washing had no significant effect on larval motility at 14 days following harvest with a marginal mean of 68.6% (95% CI 59.1–78.1%) washed in water vs. 63.3% (95% CI 53.8–72.9%) when incubated in betadine, after adjusting for larval batch (*p* = 0.38).

Larvae washed in sterile water alone did not meet bioburden criteria with overgrowth of coliform bacteria on all plates when tested on the day of preparation. Incubation in 10% and 1% betadine for 10 min resulted in acceptable microbial bioburden levels on the day of processing. Five of six cultures incubated in 1% betadine continued to meet criteria at 48 h. In the batch that failed, an overgrowth of coliforms was observed in association with evidence of larval migration on the plates, implying that the larvae had not been successfully separated from the solution. Samples tested at 7 days post-processing did not meet criteria (Table 1).

**Table 1:** The effect of post-harvest washing, incubation in antiseptic and storage in gentamicin on reducing microbial bioburden on the day of harvest and at 48 h and 7 days following harvest

Post-harvest processing	Number of experiments that met criteria/number experiments		
	Harvest day	48 h	7 days
Sterile water wash	0/3	0/1	Not performed
Betadine 10% 10 min	7/7	Not performed	Not performed
Betadine 1% 10 min	16/16	5/6	0/2
		(Larvae in sample)	
Betadine 0.1% 10 min	1/1	0/1	Not performed
Betadine 1% 10 min + 1 mg/ml gentamicin	1/1	1/1	2/2
Betadine 1% 10 min + 0.5 mg gentamicin	Not performed	Not performed	1/1
Betadine 1% 10 min + 0.1 mg/ml gentamicin	Not performed	Not performed	1/1
Betadine 1% 10 min + 0.01 mg/ml gentamicin	Not performed	Not performed	0/1
Betadine 1% 10 min + 0.001 mg/ml gentamicin	Not performed	Not performed	0/1
Betadine 1% 10 min, pre-treatment of faeces with amphotericin B and gentamicin	10/10	Not performed	0/2
Betadine 1% 10 min, no pre-treatment of faeces with amphotericin B and gentamicin	7/7	4/5	Not performed
		(Larvae in 1 sample)	

**Fig. 1:**
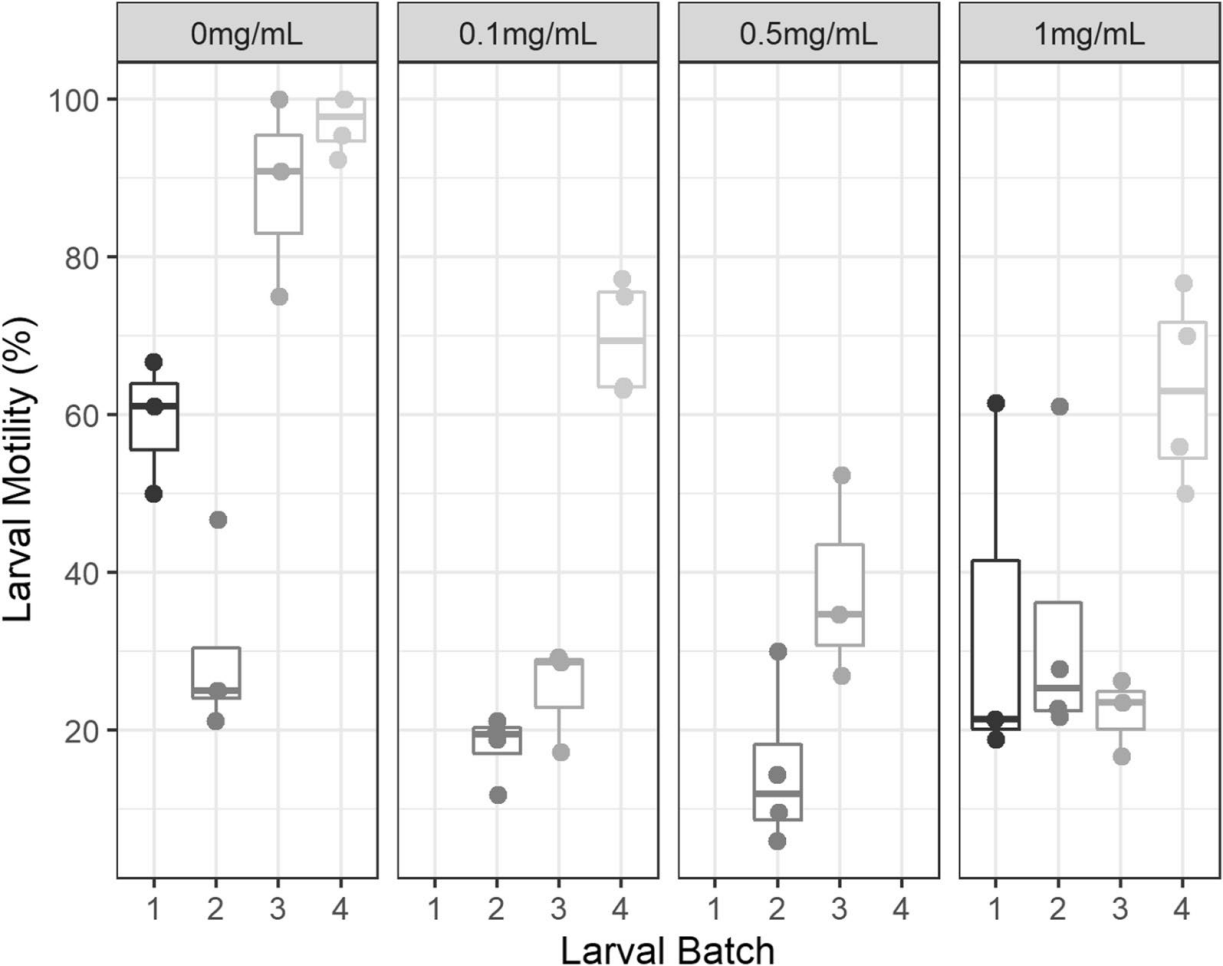
Effect of storage in gentamicin solution on larval viability assessed by thermally induced motility at 7 days post-harvest. Experiments performed on larvae from matching batches, indicated by batch number. (Dots, mean of experiment. Line, median, 95% CI)

The addition of gentamicin at doses > 0.1 mg/ml produced solutions that met bioburden criteria at day 7. However, any gentamicin treatment was found to result in significantly lower larval motility after 7 days, with marginal mean motility of combined gentamicin-treated larvae of 37.8% (95% CI 4.7–70.9%) vs. 67.3% (95% CI 35.2–99.3%, *p* < 0.001) for untreated larvae, after adjusting for batch effects (Fig. 1).

## Discussion

Despite the significant interest in experimental human hookworm infection, details of larval production methods and the larvae prepared are lacking.

In this report we have detailed the methods used to produce larvae from a single experimentally infected donor for use in a clinical trial. Although the donor inoculation occurred > 12 months prior to the commencement of this study, there was a suggestion that the faecal hookworm DNA content increased marginally over the duration of this study. Furthermore, despite the use of a single donor and standardised laboratory techniques, we found that the larval yield varied greatly between cultures (median 23.69 larvae per gram IQR 2.5–32.08) and had no relationship to the faecal hookworm DNA content. This has implications for planning the donor populations required for future clinical trials. While Hoogerwerf et al. have undertaken a detailed analysis of egg output after experimental infection, analysis of larvae recovered per gram of faeces was not reported [28]. It is possible that host and parasite factors influence egg fertility and therefore larval yield by coproculture. Intriguingly, in a human hookworm vaccine study, Chapman et al. demonstrated that significantly fewer larvae per gram were recovered at culture from vaccinated individuals compared to controls despite there being no significant difference in faecal DNA content [15]. Further investigation of the observed discrepancy between faecal egg content and larval production is indicated, given its implications for clinical trial planning and also potential novel hookworm control strategies.

It has been shown that conditions of larval culture such as pH and temperature and storage conditions have implications for larval longevity [29] and that larval infectivity diminishes with age [30]. Despite this, the reporting of culture methods, larval age and viability at the time of use has been in human trails has been sparse.

In this report we demonstrate that the viability of larvae decreases over time from a mean of 85% to < 70% within 14 days. The age of the larvae used in clinical trials is likely to be a crucial variable and perhaps may explain some of the inconsistencies in reported studies. Chapman et al. reported that a dose of 30 L3 produced patent infections in 100% of individuals 10 weeks following inoculation with larvae used within 48 h of harvest [15]. Similarly, Hoogerwerf et al. reported 100% of participants developed patent infections in 8 weeks after inoculation with larvae that were < 10 days old. In another study undertaken by Diemert et al., only 30% of participants developed patent infection after exposure to 25 L3 whereas 90% of participants developed patent infections after a dose of 50 L3. In addition, egg counts were modest and patency only developed after 100 days in some individuals. Although the age of the larvae is not reported, this study used larvae imported internationally, which were likely significantly older than those described above [19]. It is important that future clinical trials report the age of the larvae at time of use. Additionally, point-of-use assays should be developed to assess larval viability immediately prior to use.

All human trials have used larvae produced by coproculture. It is intuitively obvious that larvae produced by faecal culture are contaminated with enteric bacteria. Assessment of bioburden has been described in two recent studies. Diemert et al. [19] report the use of larvae produced by GMP method at the Immune Modulation Research Group (IMRG) at the University of Nottingham. Faeces were pre-treated with antifungal and antimicrobial agents prior to mixing with charcoal and Harada-Mori coproculture. After harvest the larvae are repeatedly washed in sterile water after which batch testing for microbial bioburden assessment was performed according to both USP 61/62 and European Pharmcopoeia 26.12 and 26.13 criteria [19]. Hoogerwerf et al. [28] report a similar method of pre-treatment and culture. However, following harvest the larvae were incubated in antiseptic prior to washing. Microbial bioburden assessment was limited to exclusion of pathogenic bacteria [31]. In this study we found that washing in sterile water was insufficient to reduce bioburden sufficiently to meet regulatory criteria. Although antiseptic treatment successfully reduced the bioburden for 48 h, recrudescence of the bacterial burden occurred within days. Furthermore, we found that storage of the larvae in antimicrobial concentrations sufficient to maintain the bioburden below levels specified by regulatory agencies was toxic to the larvae, a finding previously described by Harada [32].

The most comprehensive description of hookworm culture techniques is provided by Miller in describing his experience in developing a commercial canine hookworm vaccine. Unsurprisingly, larvae produced by coproculture methods were found to be contaminated with enteric organisms. Despite disinfection procedures, the bioburden of larvae produced by this method could not be reduced sufficiently to comply with regulatory standards and the development of a “faeces free” methodology was required to meet contemporaneous regulatory standards [33]. It is possible that future human trials may require the development of similar methodologies.

This study has limitations. A single experimentally infected donor was used for this work. It is likely that most clinical trials will be facilitated by a pool of donors and that individual variations in egg and larval yield will be less important. For logistical reasons, we did not perform microscopic evaluation of faecal egg counts. Although microscopy is well established, its sensitivity is inferior to molecular methods [34] and microscopic assessment must be performed promptly as sensitivity deteriorates within hours of stool production [35, 36].

To facilitate future human hookworm infection studies methods for GMP manufacture of larvae, storage and viability at time of use will need to be developed. We advocate that details of hookworm culture and viability assessments are described in detail and larval age and viability at the time of use be specified. Of crucial importance for experimental reproducibility is the characterisation of larval investigational products including viability over time, infectivity and the development of point-of-use assessments of viability. Furthermore, faeces-free culture methodologies should be pursued and perfected, as described by Miller [33], as well as cryopreservation and reanimation methods.

## Supplementary Information

**Additional file 1:** Stool DNA extraction and qPCR. **Figure S1.** A. Faecal hookworm PCR Cq values correlated to larval yield and over 16 months. B. Faecal hookworm PCR Cq values over time.
