# Supplementary material for: The production of Necator americanus larvae for use in experimental human infection
Source: Parasit Vectors. 2022 Jul 8;15:242. doi: 10.1186/s13071-022-05371-y (PMC9264692; doi:10.1186/s13071-022-05371-y)
Supplement: Supplementary file 1 — Additional file 1: Stool DNA extraction and qPCR. Figure S1. A. Faecal hookworm PCR Cq values correlated to larval yield and over 16 months. B. Faecal hookworm PCR Cq values over time. [file 13071_2022_5371_MOESM1_ESM.docx]

Additional file 1.

Methods S1.

**Stool DNA extraction and qPCR**

Faecal samples were washed as previously described[1]. DNA extraction was performed using the Maxwell RSC PureFood GMO and Authenitication kit with CTAB buffer (Promega, Madison, Wisconsin, USA) using Protocol 4B: Meat Sample Lysis, with modifications. Briefly samples consisted of 0.2 g faeces, to which1g Zirconia/Silica 0.5 mm beads (Daintree Scientific, St.Helens, Tasmania AUS) and 600 µL of the CTAB buffer (with added 1% w/v PVP) were added, with samples placed in a sample homogenizer for 1min. Following homogenisation, samples were placed in a 90°C dry bath for 10 min then placed at 2-8°C for 20 min. Samples were centrifuged at 10,000g for 1min with supernatant removed and placed into new 1.7ml microtube, to which 2µL of RNase A solution and 30µL of Proteinase K solution were added. Samples were placed in a 60°C dry bath for 2hrs, vortexing 2-3 times. Samples were centrifuged at 16,000xg for 10 min and supernatant transferred to the Maxwell RSC reagent cartridge. Remaining processing was as per the provided protocol.

The PCR reaction mixture consisted of GoTaq 2x Master Mix (Promega), 3.5mM MgCl_2_ (Bioline(Aust)), 2 µL of template DNA, optimized primer and probes as follows: 200nM EHV forward and reverse primers, 100nM for Ancylostoma and Necator forward and reverse primers, and 60nM for Ascaris forward and reverse primers, and 100nM for all probes. Reactions were made up to a volume of 20 μl using PCR-grade water. Amplification was performed using the following conditions: 3 minutes at 95 ˚C followed by 40 cycles of 95˚C for 9 seconds and 61˚C for 60 seconds. PCRs were run on a Bio-Rad CFX 384.

Figure S1.

Figure S1. A. Faecal hookworm PCR Cq values correlated to larval yield and over 16 months. B. Faecal hookworm PCR Cq values over time


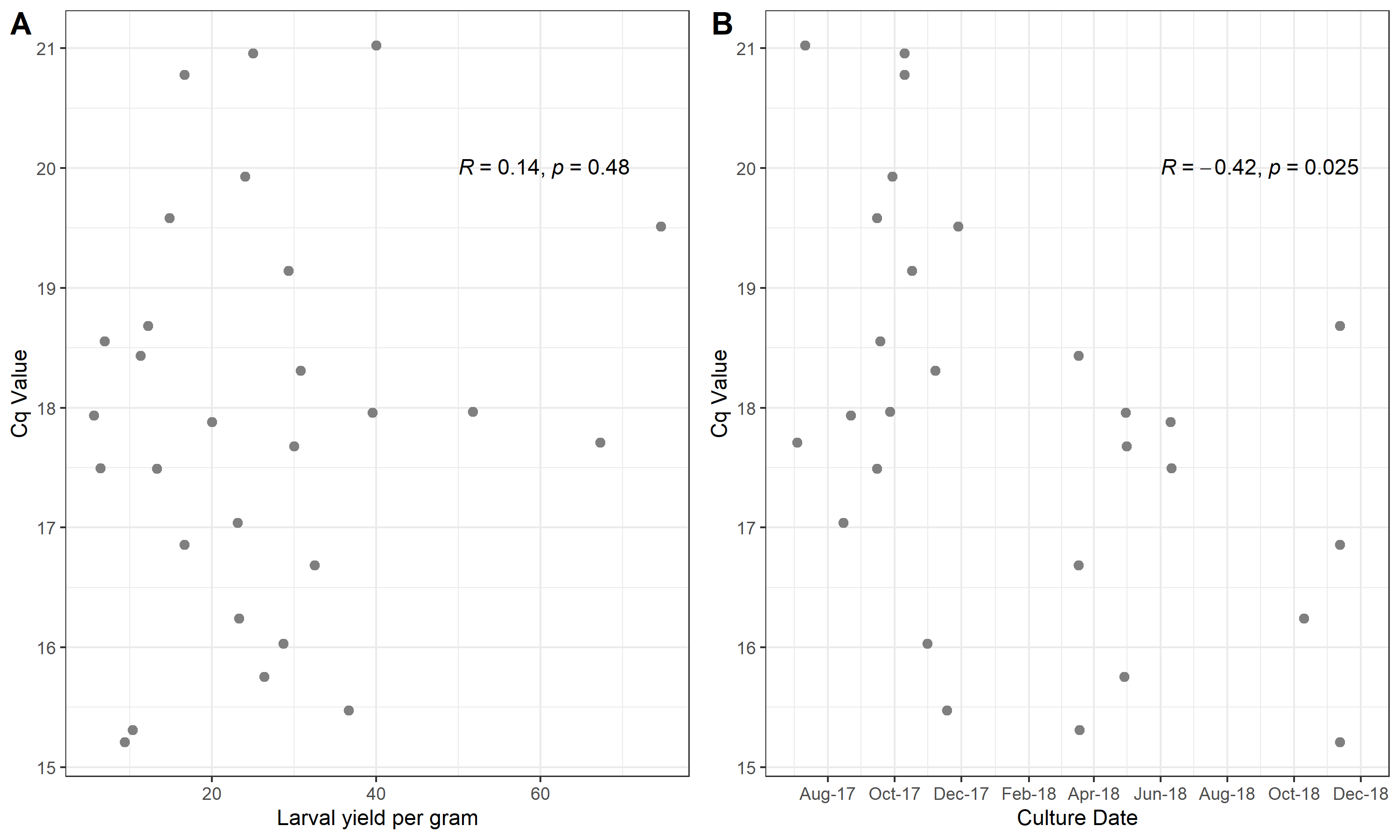


References

1. Llewellyn S, Inpankaew T, Nery SV, Gray DJ, Verweij JJ, Clements AC, et al. Application of a Multiplex Quantitative PCR to Assess Prevalence and Intensity Of Intestinal Parasite Infections in a Controlled Clinical Trial. PLoS neglected tropical diseases. 2016;10(1):e0004380. Epub 2016/01/29. doi: 10.1371/journal.pntd.0004380. PubMed PMID: 26820626; PubMed Central PMCID: PMCPMC4731196.
